# Primulagenin A is a potent inverse agonist of the nuclear receptor RAR-related orphan receptor gamma (ROR*γ*)

**DOI:** 10.1016/j.apsb.2026.03.003

**Published:** 2026-03-14

**Authors:** Patrik F. Schwarz, Alexander F. Perhal, Teresa Preglej, Lina Breit, Klaus G. Schmetterer, Ulrike Grienke, Jasmin Janneschütz, Ya Chen, Natacha Rochel, Johannes Kirchmair, Nina Schützenmeister, Judith M. Rollinger, Michael Bonelli, Verena M. Dirsch

**Affiliations:** a Department of Pharmaceutical Sciences, Division of Pharmacognosy, Faculty of Life Sciences, University of Vienna, Vienna 1090, Austria; b Vienna Doctoral School of Pharmaceutical, Nutritional and Sport Sciences (PhaNuSpo), University of Vienna, Vienna 1090, Austria; c Department of Medicine III, Division of Rheumatology, Medical University of Vienna, Vienna 1090, Austria; d Department of Laboratory Medicine, Medical University of Vienna, Vienna 1090, Austria; e Department of Pharmaceutical Sciences, Division of Pharmaceutical Chemistry, Faculty of Life Sciences, University of Vienna, Vienna 1090, Austria; f Institute of Genetics and Molecular and Cellular Biology (IGBMC), INSERM, U1258/CNRS, UMR 7104/Univ. Strasbourg, Illkirch 67404, France; g Christian Doppler Laboratory for Molecular Informatics in the Biosciences, Department for Pharmaceutical Sciences, University of Vienna, Vienna 1090, Austria

**Keywords:** Primulagenin A, Primulae radix, Natural products, Triterpenoids, ROR*γ* (ROR gamma), Th17 cells, IL-17A, Structure–activity relationship

## Abstract

Throughout history, herbal medicines and natural products have played a crucial role as therapeutics for humans, yet their molecular mechanisms of action often remain elusive. Here, we investigate whether primulagenin A (PGA) from the traditionally used herbal substance Primula root acts *via* the nuclear receptor ROR*γ*, a key regulator of pro-inflammatory Th17 cells, which are linked to autoimmune diseases like psoriasis. Full-length luciferase assays revealed a high potency (IC_50_ = 119 nmol/L) and efficacy (*I*_max_ = 87%) of PGA as an inverse agonist of ROR*γ*. To ensure sufficient supply, we established methods to isolate and synthesize PGA. Its binding to the human ROR*γ* ligand binding domain was confirmed by nano differential scanning fluorimetry, and a structure–activity relationship was proposed by docking and site-directed mutagenesis. qPCR revealed PGA-mediated downregulation of ROR*γ* target gene expression. Furthermore, PGA inhibited murine and human Th17 differentiation in a concentration-dependent manner and reduced the proportion of IL-17A-producing Th17 cells, as assessed by flow cytometry. In this work, we identify PGA as a new, potent, and efficacious inverse agonist of ROR*γ*, with potential for modulating immune responses in inflammatory and autoimmune diseases.

## Graphical abstract

The natural product PGA, isolated from *Primula* sp. and chemically synthesized, is a highly potent inverse agonist of the nuclear receptor ROR*γ* that effectively inhibits proinflammatory Th17 cell differentiation.

**Figure:**
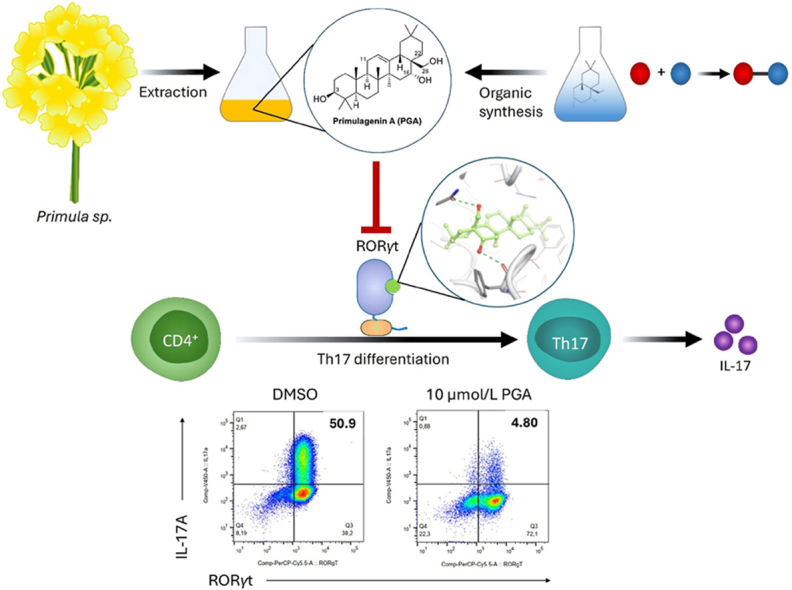

## 1 Introduction

For centuries, humans have relied on medicinal plants as a fundamental source of therapeutic compounds^1^. The importance of “unlocking nature's pharmacy” is underpinned by the number of newly approved drugs that are either natural products, derived from natural products, or “defined mixtures” that sum up to 23.5% of all drugs approved between 01/1981 and 09/2019^2^. Prominent examples of FDA-approved natural products include digoxin^3^, morphine^4^, and colchicine^5^. In contrast to these well-characterized molecules, the precise molecular mechanism of many other purified natural products is hard to pin down, even if cellular (*e.g*., antiproliferative) effects appear promising^6^. When it comes to herbal medicinal products or herbal substances, there is often a strong contrast between their successful use for centuries and the elusiveness of their pharmacological mode of action^7^^,^^8^. For instance, the rhizomes and roots of cowslip (*Primula veris* L.) or oxlip (*Primula elatior* (L.) Hill)—known as “Primula root” or “*Primulae radix*”—are traditionally used to treat cough and cold. In Europe, several herbal preparations containing Primulae Radix in liquid and solid dosage forms are marketed as herbal medicinal products against respiratory infections. Still, studies providing a solid rationale for this use are scarce^9^. In 2005, Nauert et al*.* reported that a fluid extract from Primulae radix concentration-dependently reduced the lipopolysaccharide-induced interleukin (IL)-8 release from primary monocytes. In contrast, a fluid extract from thyme hardly affected the release of this cytokine (reviewed in Ref. 10). Interestingly, clinical trials examining the effects of fixed combinations of both herbal substances found favorable effects for treating acute bronchitis^11^, ^12^, ^13^. Despite these promising findings, the molecular target remained unknown.

The RAR-related orphan receptors (RORs) are a subfamily of nuclear receptors (NRs) consisting of ROR*α*^14^, ROR*β*^15^, ROR*γ*^16^, and various isoforms of these proteins^17^. In 2006, ROR*γ*t, the ROR*γ* isoform exclusively expressed in cells of the immune system, was identified as the crucial transcription factor driving the differentiation of naïve CD4^+^ T cells into pro-inflammatory T helper (Th)-17 cells^18^. Th17 cells possess a dichotomous nature. They secrete cytokines like IL-17A, IL-17F, and IL-22 and act as host defenders against bacterial and fungal infections. Furthermore, they play a significant role in autoimmune diseases like psoriasis, rheumatoid arthritis (RA), inflammatory bowel disease (IBD), and multiple sclerosis (MS)^19^. As ROR*γ*t is an important transcription factor for Th17 cell differentiation and function of differentiated Th17 cells^20^^,^^21^, inhibition of this NR was brought up as a potential therapy option for these autoimmune diseases^22^. Depending on whether ROR*γ* is considered unliganded or liganded inside cells, ROR*γ* inhibitors can be classified as inverse agonists or antagonists, respectively^23^. Given that apo-ROR*γ* has demonstrated the capacity to adopt an active conformation in the absence of any ligands, ROR*γ* inhibitors will be referred to as inverse agonists in this study^24^. Examples of such inverse agonists range from synthetic compounds like SR2211^25^ to a myriad of natural products, including triterpenoids like ursolic acid (UA) and oleanolic acid (OA)^26^. Interestingly, *Primula elatior*, specifically, contains oleanane-type triterpene saponins that are hydrolyzed to primulagenin A (PGA) under acidic conditions^9^^,^^27^^,^^28^. Here, we present PGA as a new and highly active inverse agonist of ROR*γ*, highlighting its promise for therapeutic immunomodulation through inhibition of the pro-inflammatory Th17 response.

## 2 Materials and methods

### 2.1 Materials

All purchased items including companies and identifiers can be found in Supporting Information Table S1–S3. Gifted plasmids and all used primers are listed in Supporting Information Tables S4 and S5, respectively. All self-designed primers were purchased from Microsynth (Balgach, Switzerland).

### 2.2 Compound stocks, dilutions, and identity/purity checks

Compounds were dissolved in 100% DMSO under sterile conditions (HERAsafe KS18, Thermo Fisher Scientific, Waltham, MA, USA). Compound stocks and dilutions were stored at −70 °C until use. For identity and purity checks, these stock solutions were diluted 1:100 with MeOH before analysis.

Compound identity and purity were analyzed using a UHPLC–DAD-CAD–MS system. The setup consisted of an Ultimate 3000 UHPLC system (Thermo Fisher Scientific, San Jose, CA, USA) coupled to DAD and CAD detectors, using a reversed-phase ACQUITY UPLC CSH C18 column (130 Å, 1.7 μm, 2.1 mm × 100 mm; Waters Corp., Framingham, MA, USA). Mobile phases were A: H_2_O with 0.01% formic acid, and B: acetonitrile; both were degassed prior to use. A 10-min binary gradient was applied at a flow rate of 350 μL/min: 0–1 min, 80% B; 2–6 min, 80%–98% B; 6–8 min, 98% B; 8–10 min, re-equilibration with 80% B. Samples (10 μL, in MeOH) were injected, followed by a blank to ensure column cleaning and re-equilibration. Purity was assessed *via* DAD and CAD chromatograms. Compound identity was confirmed by mass spectrometry using an LTQ-XL linear ion trap mass spectrometer (Thermo Fisher Scientific, San Jose, CA, USA) with a HESI source (capillary temp: 350 °C; sheath/aux/sweep gas: 54/12/3 arbitrary units; spray voltage: 3.5 kV). Data were acquired in both positive and negative ion modes over an *m*/*z* range of 100–2000 Da.

### 2.3 Plant material

A mixture of dried cut roots of two *Primula* species (batch No. P21301312, Primulae Radix), *i.e.*, *Primula veris* L. and *Primula elatior* (L.) Hill (Primulaceae), was obtained from Kottas Pharma GmbH (Eitnergasse 8, 1230 Vienna, Austria) in 2021. The material was authenticated by Kottas Pharma GmbH according to the monograph of the European Pharmacopoeia. A voucher specimen (JR-20210422_A1) is deposited in the Herbarium of the Department of Pharmaceutical Sciences, Division of Pharmacognosy, University of Vienna, Austria.

### 2.4 Cell culture

Cell culture techniques were performed under sterile conditions. All incubation steps were performed at 37 °C and 5% CO_2_. Human embryonic kidney (HEK)293 and EL-4 (mROR*γ*t) cells were cultured in DMEM, supplemented with 10% fetal bovine serum (FBS), 2 mmol/L l-glutamine, and Penicillin–Streptomycin (100 U/mL penicillin and 100 μg/mL streptomycin) (“complete DMEM”). Jurkat T and HepG2 cells were cultured in RPMI 1640 and EMEM containing the same supplements, respectively. All media contained phenol red. Cells were subcultured every 2–3 days and only used up to in-house passage number of 30. Cell counting and viability were checked using a Vi-CELL XR Cell Viability Analyzer (Beckman Coulter, Krefeld, Germany). Where indicated, 5% charcoal-stripped FBS replaced 10% FBS (“stripped” media). Where indicated, stripped DMEM without phenol red was used.

### 2.5 Luciferase reporter gene assay

8 × 10^6^ HEK293 cells in complete DMEM were seeded onto a 150 mm dish and incubated for 5 h. Afterwards, cells were transfected with 5 μg of a plasmid encoding the NR, 5 μg of a plasmid encoding the luciferase reporter including the respective response element, and 3 μg of a plasmid encoding eGFP using the calcium phosphate co-precipitation method^29^. After overnight incubation, medium was changed to complete DMEM. After 4–5 h, cells were trypsinized (0.05% trypsin +537 μmol/L Na_2_-EDTA·2H_2_O in 1000 mL PBS), resuspended in stripped DMEM and seeded into a 96-well plate at a density of 5 × 10^4^ cells per well. Cells were treated with the vehicle control, the positive control, or the compounds of interest and incubated for 18 h. Afterwards, cells were checked microscopically for signs of toxicity or compound precipitation. Medium was removed and cells were frozen at −70 °C for at least 1 h. Cells were lysed with the Reporter Lysis 5 × Buffer enriched with coenzyme A (CoA; 450 μmol/L) and dithiothreitol (DTT; 5 mmol/L). Relative fluorescence unit (RFU) and relative luminescence unit (RLU) values were measured on a Tecan Spark (Tecan Group, Männedorf, Switzerland), the latter after applying 50 μL adenosine triphosphate (ATP) and D-luciferin *via* injectors A and B, respectively. The RLU/RFU ratio was calculated to account for differences in cell number and transfection efficiency. RLU/RFU values were then normalized to the vehicle control and are expressed as “fold activation”.

### 2.6 General experimental procedures connected to the isolation of PGA

Normal phase flash chromatography was performed on an Interchim puriFlash 4250 system (Interchim, Montluçon, France), equipped with an evaporative light scattering detector (ELSD), a photodiode array (PDA) and a fraction collector, controlled by Interchim Software. A PuriFlash Silica HP column (15 μm, 120 g) served as stationary phase and the mobile phase consisted of *n*-hexane, acetone, and methanol. Semi-preparative supercritical fluid chromatography (SFC) was performed on a Waters Prep-15 System equipped with an ELSD, a PDA, and a fraction collector. A Waters Viridis Prep BEH column (5 μm; 10 mm × 250 mm) served as the stationary phase and data were analyzed using MassLynx (Waters Corp.). The mobile phase consisted of a supercritical CO_2_/organic modifier (MeOH) gradient (temperature, 45 °C; flow rate, 15 mL/min). The fractions obtained from all chromatographic steps were analyzed by TLC (mobile phase: CH_2_Cl_2_–acetone, 5.5:1); stationary phase: Merck silica gel 60 PF_254_ (Merck KGaA, Darmstadt, Germany) detected after derivatization with vanillin/H_2_SO_4_ (5% in MeOH) under both visible light and UV_254_ and UV_366_. Ultra-high-performance supercritical fluid chromatography (UHPSFC) was carried out using an Acquity UPC^2^ (ultra-performance convergence chromatography; Waters Corp.) instrument comprising a sample-, binary solvent-, column-, isocratic solvent- and convergence manager with a PDA detector and a Quadrupole Dalton (QDa) MS detector equipped with electrospray ionization (ESI) in the positive and negative modes: capillary voltage, 0.8 kV; nebulizer, 0.4 bar (N_2_); dry gas flow, 4 L/min (N_2_); probe temperature, 500 °C. The instrument was controlled *via* Empower 3 software (Waters Corp.).

1D and 2D NMR experiments were performed by using a Bruker Avance 500 NMR spectrometer (UltraShield) with a 5 mm switchable probe (TCI Prodigy CryoProbe, 5 mm, triple resonance inverse detection probe head) with *z* axis gradients and automatic tuning and matching accessory (Bruker Corp., Billerica, MA, USA). The sample (∼2 mg) were measured at 298 K in fully deuterated CDCl_3_ referenced to the residual non-deuterated solvent signals. The resonance frequency for ^1^H NMR was 500.13 MHz and for ^13^C NMR 125.75 MHz. Standard 1D and gradient-enhanced (ge) 2D experiments, like HSQC, HMBC, and NOESY were used as supplied by the manufacturer.

### 2.7 Extraction, isolation, and identification of PGA

The dried cut roots of *Primula* sp. (∼1 kg) were macerated with 3 L MeOH (at 22 °C, three times for 48 h each). After removal of the solvent under vacuum, the extract was reconstituted in a mixture of MeOH and water (1:1) and separated *via* liquid–liquid partition using petroleum ether. The combined aqueous MeOH phases were then acidified by adding concentrated HCl to reach a pH value of 1.5. To hydrolyze the contained saponins, this mixture was stirred and heated at 95 °C for several h. Successful hydrolysis of saponins was confirmed *via* TLC. The obtained extract (PRA) was partitioned by CH_2_Cl_2_ to separate aglycones (PRA_A), *i.e.*, sapogenins (in the CH_2_Cl_2_ phase) from sugars (PRA_S) in the remaining aqueous phase. The PRA_A fraction (13 g) was then subjected to normal phase flash chromatography resulting in eight fractions (1.1 to 1.8). Fraction 1.4 (890 mg) was further separated using Sephadex LH 20 column chromatography (mobile phase: CH_2_Cl_2_–acetone, 85:15) to give four fractions (2.1 to 2.4). Fraction 2.4 (270 mg) was then subjected to semi-preparative SFC resulting in seven fractions (3.1 to 3.7). Fraction 3.5 was identified as primulagenin A (PGA, 20 mg). The purity of PGA was determined to be 97.4% by ^1^H NMR^30^.

### 2.8 Organic synthesis of triterpenoids

#### 2.8.1 General information

All reactions requiring anhydrous conditions were performed under argon atmosphere in oven dried glassware applying *Schlenk* technique. Reactions carried out at 0 °C employed an ice bath. All chemicals were purchased from Thermo Fisher Scientific or Sigma–Aldrich (St. Louis, MO, USA) and used as received, unless otherwise noted. THF was dried by storing it over 4 Å molecular sieves under argon atmosphere. Infrared spectra were obtained from neat solids or liquids on a Bruker Alpha with a Platinum-ATR unit. Melting points were determined in open capillary tubes on a Cole-Parmer Stuart SMP11 melting point apparatus (Antylia Scientific, Vernon Hills, IL, USA) and are uncorrected. Optical rotations were measured using an Anton Paar MCP 100 polarimeter (Anton Paar GmbH, Graz, Austria). Concentrations (*c*) of specific rotations are given in g/100 mL. High resolution mass spectra (HRMS) were measured with a Bruker maXis HD ESI-QTOF. NMR spectra were recorded on a Bruker AVANCE III ASCEND 400 MHz spectrometer with BBFO-PLUS probe or on Bruker AVANCE III HD Ultrashield 500 MHz spectrometer with TCI H/F-C-N Prodigy Kryo-probe head at 298 K. As internal standard, the residual signal of deuterated chloroform 7.26/77.16 ppm (*δ* ^1^H/^13^C ppm) was used^31^. All relevant signals are listed as follows: chemical shift, multiplicity (s = singlet, d = doublet, dd = doublet of doublets, ddd = doublet of doublet of doublets, t = triplet, td = triplet of doublets, m = multiplet), coupling constant(s) and number of protons/carbons. Reactions were monitored by thin layer chromatography (TLC) on silica-coated aluminum plates (DC Kieselgel Alugram® Xtra SIL G/UV254, layer thickness 0.2 mm; Macherey-Nagel, Düren, Germany). Visualization was performed with vanillin staining (6.0 g vanillin, 1.5 mL sulfuric acid >95%, 95 mL ethanol). Flash column chromatography was performed using silica 60 (particle size 0.040–0.063 nm, 230–400 mesh, Macherey-Nagel) at room temperature using a MPLC-system (Biotage® Selekt; Biotage AB, Uppsala, Sweden).

#### 2.8.2 Synthesis of primulagenin A

Under argon-atmosphere, lithium aluminum hydride (LiAlH_4_) (powder, 76.3 mg, 2.01 mmol, 3.80 equiv.) was added in portions at 0 °C to a solution of echinocystic acid (250 mg, 529 μmol, 1.00 equiv.) in anhydrous THF (60 mL). The reaction mixture was heated under reflux for 3 h, cooled to room temperature and stirred at this temperature for 18 h. Afterwards, the reaction mixture was cooled to 0 °C, quenched by the successive addition of water (10 mL), 4 mol/L aqueous NaOH (10 mL) and water (10 mL) and warmed to room temperature within 15 min. Subsequently, the reaction mixture was diluted with methyl *tert*-butyl ether (MTBE) (30 mL) and washed with sat. aqueous NaHCO_3_-solution (30 mL), followed by the extraction of the aqueous phase with MTBE (3 × 30 mL). The combined organic phases were washed with brine (60 mL), dried over MgSO_4_, filtered and the solvent was removed *in vacuo*. The obtained residue was purified by column chromatography (SiO_2_, CH_2_Cl_2_/MeCN 9:1  →  5:1) to yield primulagenin A (210 mg, 457 μmol, 86%) as white solid.

TLC: *R*_f_ = 0.30 (CH_2_Cl_2_/MeCN 6:1). Mp.: 234 °C. ${\left[\alpha \right]}_{D}^{20}$: +24.0 (*c* = 0.10, CHCl_3_). IR (ATR): *ṽ* (cm^−1^) = 3888, 2934, 2157, 1457, 1382, 1337, 1301, 1258, 1192, 1080, 1038, 998, 727, 656, 631. ^1^H NMR (400 MHz, CDCl_3_): *δ* (ppm) = 0.73–0.77 (m, 1H, 5-H), 0.79 (s, 3H, 24-H_3_), 0.92 (s, 6H, 29-H_3_, 30-H_3_), 0.93 (s, 3H, 26-H_3_), 0.94 (s, 3H, 25-H_3_), 0.95–0.99 (m, 1H, 1-H_A_), 1.00 (s, 3H, 23-H_3_), 1.08 (ddd, *J* = 1.2, 3.8, 12.4 Hz, 1H, 19-H_A_), 1.24–1.28 (m, 1H, 21-H_A_), 1.34 (s, 3H, 27-H_3_), 1.34–1.40 (m, 1H, 15-H_A_), 1.38–1.43 (m, 2H, 6-H_A_, 7-H_A_), 1.52–1.56 (m, 1H, 7-H_B_), 1.54–1.60 (m, 2H, 6-H_B_, 9-H), 1.57–1.62 (m, 1H, 21-H_B_), 1.59–1.64 (m, 4H, 2-H_2_, 22-H_2_), 1.62–1.66 (m, 1H, 1-H_B_), 1.86–1.90 (m, 2H, 11-H_2_), 1.89–1.94 (m, 1H, 15-H_B_), 1.91–1.96 (m, 1H, 18-H), 2.04–2.10 (m, 1H, 19-H_B_), 3.22 (dd, *J* = 4.7, 11.1 Hz, 1H, 3-H), 3.32 (s, 2H, 28-H_2_), 4.05 (t, *J* = 4.8 Hz, 1H, 16-H), 5.32 (t, *J* = 3.7 Hz, 1H, 12-H). ^13^C NMR (126 MHz, CDCl_3_): *δ* (ppm) = 15.7 (C-24), 15.9 (C-25), 17.3 (C-26), 18.4 (C-6), 23.5 (C-11), 25.6 (C-30), 26.4 (C-22), 27.4 (C-2), 27.4 (C-27), 28.2 (C-23), 30.5 (C-20), 32.9 (C-7), 32.9 (C-29), 34.9 (C-15), 35.4 (C-21), 37.1 (C-10), 38.8 (C-1), 38.9 (C-4), 40.1 (C-8), 40.7 (C-17), 41.7 (C-14), 42.8 (C-18), 47.1 (C-9), 47.1 (C-19), 55.4 (C-5), 70.9 (C-28), 75.1 (C-16), 79.1 (C-3), 122.9 (C-12), 143.1 (C-13). HRMS (ESI^+^) = *m*/*z* calcd.: 481.3652 C_30_H_50_NaO_3_^+^; found: 481.3650. C_30_H_50_O_3_ (458.72 g/moL).

#### 2.8.3 Synthesis of erythrodiol

Under argon-atmosphere, LiAlH_4_ (powder, 190 mg, 4.99 mmol, 3.80 equiv.) was added in portions at 0 °C to a solution of oleanolic acid (600 mg, 1.31 mmol, 1.00 equiv.) in anhydrous THF (30 mL). The reaction mixture was heated to reflux for 3 h, cooled to room temperature and stirred at this temperature for 16 h. Afterwards, the reaction mixture was cooled to 0 °C, quenched by the successive addition of water (10 mL), 4 mol/L aqueous NaOH (10 mL) and water (10 mL) and warmed to room temperature within 15 min. Subsequently, the reaction mixture was diluted with methyl *tert*-butyl ether (MTBE) (40 mL) and was washed with water (2 × 30 mL), followed by the extraction of the aqueous phase with MTBE (3 × 30 mL). The combined organic phases were washed with brine (50 mL), dried over MgSO_4_, filtered and the solvent was removed *in vacuo*. The obtained residue was purified by column chromatography (SiO_2_, PE/EtOAc 19:1  →  3:1) to yield erythrodiol (400 mg, 903 μmol, 69%) as white solid.

TLC: *R*_f_ = 0.34 (PE/EtOAc 4:1). Mp.: 224 °C. ${\left[\alpha \right]}_{D}^{20}$: +72.5 (c = 0.10, CHCl_3_). IR (ATR): *ṽ* (cm^−1^) = 3327, 2924, 2867, 1462, 1365, 1305, 1189, 1148, 1093, 1041, 1000, 816, 716, 603, 521. ^1^H NMR (400 MHz, CDCl_3_): *δ* (ppm) = 0.72–0.75 (m, 1H, 5-H), 0.79 (s, 3H, 24-H_3_), 0.87 (s, 3H, 30-H_3_), 0.89 (s, 3H, 29-H_3_), 0.93 (s, 3H, 25-H_3_), 0.94 (s, 3H, 26-H_3_), 0.94–0.99 (m, 1H, 1-H_A_), 0.97–1.02 (m, 1H, 15-H_A_), 1.00 (s, 3H, 23-H_3_), 1.04–1.08 (m, 1H, 19-H_A_), 1.14–1.18 (m, 1H, 16-H_A_), 1.17 (s, 3H, 27-H_3_), 1.17–1.21 (m, 1H, 21-H_A_), 1.27–1.33 (m, 1H, 21-H_B_), 1.31–1.35 (m, 1H, 7-H_A_), 1.32–1.37 (m, 1H, 22-H_A_), 1.37–1.43 (m, 1H, 6-H_A_), 1.49–1.55 (m, 2H, 7-H_B_, 22-H_B_), 1.52–1.58 (m, 2H, 6-H_B_, 9-H), 1.56–1.63 (m, 2H, 2-H_2_), 1.60–1.64 (m, 1H, 1-H_B_), 1.67–1.73 (m, 1H, 15-H_B_), 1.70–1.75 (m, 1H, 19-H_B_), 1.83–1.90 (m, 2H, 11-H_2_), 1.86–1.93 (m, 1H, 16-H_B_), 1.96–2.00 (m, 1H, 18-H), 3.21–3.23 (m, 2H, 28-H_A_, 3-H), 3.55 (d, *J* = 10.9 Hz, 1H, 28-H_B_), 5.19 (t, *J* = 3.6 Hz, 1H, 12-H). ^13^C NMR (126 MHz, CDCl_3_): *δ* (ppm) = 15.7 (C-25), 15.7 (C-24), 16.9 (C-26), 18.5 (C-6), 22.1 (C-16), 23.7 (C-11), 23.7 (C-30), 25.7 (C-15), 26.1 (C-27), 27.4 (C-2), 28.2 (C-23), 31.1 (C-20), 31.2 (C-22), 32.7 (C-7), 33.3 (C-29), 34.2 (C-21), 37.1 (2C, C-10, C-17), 38.7 (C-1), 38.9 (C-4), 39.9 (C-8), 41.9 (C-14), 42.5 (C-18), 46.6 (C-19), 47.7 (C-9), 55.3 (C-5), 69.8 (C-28), 79.1 (C-3), 122.5 (C-12), 144.3 (C-13). HRMS (ESI^+^) = *m*/*z* calcd.: 465.3703 C_30_H_50_NaO_2_^+^ found: 465.3717. C_30_H_50_O_2_ (442.73 g/moL).

#### 2.8.4 Synthesis of oleanolic aldehyde

Under argon-atmosphere, erythrodiol (375 mg, 847 μmol, 1.00 equiv.) was dissolved in anhydrous CH_2_Cl_2_ (12 mL). Then, 2,2,6,6-tetramethylpiperidinyloxyl (TEMPO) (26.5 mg, 169 μmol, 0.20 equiv.) and bis(acetoxy)iodobenzene (BAIB) (409 mg, 1.27 mmol, 1.50 equiv.) were added to the reaction mixture and the mixture was stirred at RT for 16 h. Upon reaction control (TLC), another portion of BAIB (81.1 mg, 254 μmol, 0.30 equiv.) and TEMPO (26.5 mg, 169 μmol, 0.20 equiv.) were added, and the reaction mixture was stirred at RT for 3 h and additionally at 40 °C for 2 h. The reaction mixture was quenched with sat. aqueous NaHSO_3_-solution (5 mL) and the organic phase was washed with sat. aqueous NaHCO_3_-solution (10 mL). The aqueous phase was extracted with CH_2_Cl_2_ (3 × 10 mL) and the combined organic phases were washed with brine (25 mL), dried over MgSO_4_, filtered and the solvent was removed *in vacuo*. The obtained residue was purified by column chromatography (SiO_2_, PE/EtOAc 19:1  →  6:1) yielding oleanolic aldehyde (160 mg, 362 μmol, 43%) as white solid.

TLC: R_*f*_ = 0.52 (PE/EtOAc 4:1). Mp.: 176 °C. ${\left[\alpha \right]}_{D}^{20}$: +102.0 (c = 0.10, CHCl_3_). IR (ATR): *ṽ* (cm^−1^) = 3513, 2934, 2859, 1712, 1460, 1367, 1294, 1177, 1141, 1096, 1043, 998, 921, 752, 601. ^1^H NMR (400 MHz, CDCl_3_): *δ* (ppm) = 0.70–0.74 (m, 1H, 5-H), 0.74 (s, 3H, 26-H_3_), 0.78 (s, 3H, 24-H_3_), 0.91 (s, 3H, 25-H_3_), 0.91 (s, 3H, 30-H_3_), 0.92 (s, 3H, 29-H_3_), 0.95–0.99 (m, 1H, 1-H_A_), 0.99 (s, 3H, 23-H_3_), 1.08 (ddd, *J* = 2.5, 4.2, 13.5 Hz, 1H, 15-H_A_), 1.14 (s, 3H, 27-H_3_), 1.17–1.22 (m, 2H, 22-H_A_, 19-H_A_), 1.24–1.33 (m, 3H, 21-H_2_, 7-H_A_), 1.35–1.39 (m, 1H, 6-H_A_), 1.42–1.48 (m, 2H, 7-H_B_, 22-H_B_), 1.49–1.54 (m, 1H, 9-H), 1.51–1.55 (m, 1H, 6-H_B_), 1.55–1.58 (m, 1H, 16-H_A_), 1.57–1.63 (m, 3H, 2-H_2_, 1-H_B_), 1.62–1.67 (m, 1H, 15-H_B_), 1.66–1.70 (m, 1H, 19-H_B_), 1.85–1.90 (m, 2H, 11-H_2_), 1.98 (td, *J* = 4.2, 13.5 Hz, 1H, 16-H_B_), 2.63 (dd, *J* = 4.4, 13.8 Hz, 1H, 18-H), 3.20–3.22 (m, 1H, 3-H), 5.34 (t, *J* = 3.7 Hz, 1H, 12-H), 9.40 (s, 1H, 28-H). ^13^C NMR (126 MHz, CDCl_3_): *δ* (ppm) = 15.5 (C-25), 15.7 (C-24), 17.2 (C-26), 18.4 (C-6), 22.2 (C-16), 23.6 (C-11), 23.6 (C-30), 25.7 (C-27), 26.9 (C-15), 27.3 (C-2), 27.9 (C-22), 28.3 (C-23), 30.8 (C-20), 32.9 (C-7), 33.2 (C-29), 33.3 (C-21), 37.1 (C-10), 38.6 (C-1), 38.9 (C-4), 39.7 (C-8), 40.6 (C-18), 41.8 (C-14), 45.7 (C-19), 47.7 (C-9), 49.2 (C-17), 55.3 (C-5), 79.1 (C-3), 123.4 (C-12), 143.1 (C-13), 207.7 (C-28). HRMS (ESI^+^) = *m*/*z* calcd.: 441.3727 C_30_H_49_O_2_^+^ found: 441.3753. C_30_H_48_O_2_ (440.71 g/moL).

### 2.9 Resazurin conversion assay (cell viability assay)

For resazurin conversion assays, the different cell lines were treated with the vehicle control, the cytotoxic positive control (digitonin at 20 or 50 μg/mL), or the compounds of interest for different periods of time (in accordance with the treatment duration in the respective assay). Afterwards, the medium was carefully removed and replaced with stripped DMEM medium without phenol red containing 10 μg/mL resazurin sodium salt. Cells were incubated for 5 h and then measured on a Tecan Spark at *λ*_em_ = 590 nm. Cell viability was calculated according to Eq. (1):

(1) $$\text{Cell}\;\text{viability}\;\left(\%\right)=\frac{\text{RFU}\;\text{value}\;\text{after}\;\text{compound}\;\text{treatment}}{\text{RFU}\;\text{value}\;\text{after}\;\text{vehicle}\;\text{control}\;\text{treatment}}\times 100$$

Cytotoxicity was defined as cell viability <70% according to the ISO 10993-5 guidelines^32^.

### 2.10 Thermal stability assay

PGA and T0901317 were dissolved in absolute ethanol at a concentration of 10^−2^ mol/L. Compounds were stored at −20 °C until use.

The sequence encoding the His-hROR*γ* (264–518) ligand binding domain (LBD) was inserted into a pET15b expression plasmid. The protein was expressed in *Escherichia coli* BL21 DE3 by induction with 1 mmol/L IPTG at an OD600 of ∼0.8 and incubation at 22 °C for 150 min. Soluble proteins were purified on Ni Hitrap FFcrude column (Cytiva, Marlborough, MA, USA), followed by His tag removal by thrombin cleavage and by size exclusion chromatography on HiLoad Superdex200 column (Cytiva) equilibrated in 20 mmol/L Tris–HCl pH 7.5, 150 mmol/L NaCl, 1 mmol/L TCEP. Purity and homogeneity of the hROR*γ* LBD was assessed by SDS-PAGE. The purified protein was concentrated to 0.5–1.0 mg/mL with an Amicon Ultra 10 kDa MWCO (Merck KGaA). The aliquots of purified LBD were incubated with 4 equivalent ligands or vehicle for thermal stability experiment.

Fluorescence based thermal experiments were performed using Prometheus NT.48 (NanoTemper Technologies GmbH, München, Germany) with grade standard capillaries containing 10 μL ROR*γ* LBD with different ligands. The temperature was increased by a rate of 1 °C/min from 20 to 95 °C and the fluorescence at emission wavelengths of 330 and 350 nm was measured for 3 technical replicates. NanoTemper PR.Stability Analysis v1.0.2 was used to fit the data and to determine the melting temperatures *T*_m_. The presented data are the average of two biological replicates (one in case of T0901317).

### 2.11 Molecular docking

An X-ray crystal structure of the ROR*γ* LBD in complex with ursonic acid (PDB structure 6J3N, resolution 1.99 Å) was utilized for docking with Glide^33^, ^34^, ^35^ (part of the Schrödinger Platform, version 2023-4; Schrödinger Inc., New York, NY, USA). The protein structure was prepared with the Protein Preparation Wizard within the Maestro molecular modeling environment^36^ (part of the Schrödinger Platform) using default settings. The preparation included the (i) addition of hydrogen atoms, (ii) assignment of bond orders, (iii) assignment of protonation and metal charge states with Epik^37^^,^^38^, (iv) sampling H_2_O orientations and optimization of the hydrogen bond network, and (v) restrained minimization using the OPLS4 force field^39^ to converge heavy atoms to an RMSD of 0.30 Å. The 3D structure of PGA and OA were prepared with LigPrep^40^ using default settings including assigning ionization states at pH 7.4 ± 2.0 with Epik and optimizing geometries with the OPLS4 force field. For docking with Glide, the ligand binding site was defined within the Receptor Grid Generation wizard to dock ligands with similar size to the co-crystallized ligand. Glide Standard Precision (Glide SP) was used for ligand docking and up to 20 docking poses were set for output.

### 2.12 Site-directed mutagenesis

hGal4-ROR*γ* Q286T, H323A, and the double mutant were generated using the QuikChange Lightning Site-Directed Mutagenesis Kit according to the manufacturer's instructions. In all cases, 10 ng template (*i.e.*, hGal4-ROR*γ* for generation of the Q286T and H323A mutants or hGal4-ROR*γ* H323A for generation of the double mutant) were PCR-amplified using the mutagenic forward and reverse primers listed in Supporting Information Table S5. All mutagenic primers were designed using the QuikChange Primer Design tool (https://www.agilent.com/store/primerDesignProgram.jsp). PCR settings are shown in Supporting Information Table S6. After performing the PCR reaction, the PCR product was DpnI digested at 37 °C for 5 min and 2 μL were transformed into XL1-Blue competent *E. coli*^41^. After overnight incubation at 37 °C, a single colony was picked and added to 5 mL of lysogeny broth (LB) medium containing 50 μg/mL zeocin (“pre-culture”). After shaking for 5 h at 37 °C, the pre-culture was added to 400 mL of LB medium containing 50 μg/mL zeocin and shaken at 37 °C overnight. After centrifugation of the main culture at 5691 × *g* for 10 min and removal of the supernatant, plasmid preparation was done using the PureLink HiPure Plasmid Midiprep Kit according to the manufacturer's instructions. Plasmid purities and yields were checked using a NanoDrop 2000c (Thermo Fisher Scientific). hGal4-ROR*γ* mutants were checked for the presence of the right mutations by Sanger sequencing (Microsynth) using the sequencing primers listed in Table S5.

### 2.13 Cloning of a mRORγt-pIRES2-eGFP vector

First, mROR*γ*t was PCR-amplified out of the full-length murine ROR*γ*t vector using the Herculase II Fusion DNA Polymerase kit according to the manufacturer's instructions. For PCR-amplification, primers including a NheI (mRORgt pIRES2-eGFP NheI fwd) and a XhoI (mRORgt pIRES 2-eGFP XhoI rev) restriction site to the 5′ and 3′ end of the fragment, respectively, were used. Primers were designed using SnapGene Viewer (Dotmatics) and their sequences are listed in Table S5. PCR conditions are shown in Supporting Information Table S7. The PCR product (“fragment”) was separated on a 1% agarose gel containing 0.5X SYBR Safe and extracted using the Monarch DNA Gel Extraction Kit according to the manufacturer's instructions. After double digestion of the fragment and vector (pIRES2-eGFP) with NheI-HF and XhoI^42^, separation on a 1% agarose gel, and DNA gel extraction, ligation with a 7 × molar excess of fragment relative to vector was performed using the T4 DNA ligase^43^. Transformation and plasmid preparation were done as described in the “Site-directed mutagenesis” section with minor adaptions: 5 μL ligation product was transformed, and a different selection antibiotic (ampicillin, 50 μg/mL) was used. Sanger sequencing was employed to confirm the correct insertion of mROR*γ*t within the pIRES2-eGFP vector (primer used: “IRES-for”, see Table S5 for its sequence).

### 2.14 Generation of the stable EL-4-mRORγt cell line

The AseI-linearized^42^ mROR*γ*t-pIRES2-eGFP vector was transfected into EL-4 cells using Lipofectamine LTX according to the manufacturer's instructions. The eGFP^high^ population was sorted twice using a CytoFLEX SRT (Beckman Coulter) and cultured in complete DMEM until confluency was reached each time. Ultimately, a third sorting procedure was performed and a single eGFP^high^ cell was sorted per well in a 96-well plate containing complete DMEM enriched with 1000 μg/mL G418 for selection. The clones were allowed to grow to confluency and were later analyzed using a MACSQuant Analyzer 10 flow cytometer (Miltenyi Biotec, Bergisch Gladbach, Germany) and FlowJo 10.8.1 (BD Corp., Franklin Lakes, NJ, USA). All cells were found to be eGFP^+^ compared to WT EL-4 cells. Aliquots of these EL-4-mROR*γ*t cells were frozen and subsequently used for further experiments.

### 2.15 Quantitative reverse-transcription polymerase chain reaction (qPCR)

All steps prior to qPCR (seeding, treatment, …) for HepG2 and Jurkat T cells were done as described previously^44^. For EL-4-mROR*γ*t cells, 5 × 10^5^ cells were seeded per well of a 24-well plate in 500 μL complete DMEM, treated with compounds for 20–24 h, and stimulated with phorbol 12-myristate 13-acetate (PMA) and ionomycin using the “Cell Activation Cocktail” (PMA: 40.5 μmol/L, ionomycin: 669.3 μmol/L; 500 × ) for approx. 4.5 h. Total RNA was isolated using the innuPREP RNA Mini Kit 2.0 according to the manufacturer's instructions. 1 μg RNA was reverse-transcribed into cDNA using the High-Capacity cDNA Reverse Transcription Kit according to the manufacturer's instructions.

qPCR for all cell lines was performed using either the GoTaq Green Master Mix or the Luna Universal qPCR Master Mix on a LightCycler 480 (Roche Diagnostics). All steps were performed according to the manufacturer's instructions. Measurement setting on the LightCycler 480 and qPCR primers are listed in Supporting Information Table S8 (GoTaq)/Supporting Information Table S9 (Luna), and Table S5, respectively. qPCR primers were either bought (*G6PC*, *GAPDH*) or self-designed (rest) using Primer-BLAST (https://www.ncbi.nlm.nih.gov/tools/primer-blast/). Primer efficiencies for all self-designed primers were checked and found to be >95%. Gene expression data were first normalized to the control genes by employing the 2^−ΔΔCt^ method^45^ and then normalized to the vehicle control.

### 2.16 Murine Th17 polarization

Splenocytes were isolated from OT-II transgenic mice (B6.Cg-Tg (TcraTcrb)425Cbn/J; The Jackson Laboratory, Bar Harbor, ME, USA), and after lysis of erythrocytes with BD Pharm Lyse Lysing Buffer according to the manufacturer's instructions, stimulated with 4 μg/mL ovalbumin peptide on 48-well plates (2 × 10^6^ cells/well) in 1 mL T cell medium/well RPMI 1640 (supplemented with 10% FCS, GlutaMAX, penicillin–streptomycin, and 50 mmol/L *β*-mercaptoethanol (*β*-ME)) under Th17 polarizing conditions using 1 ng/mL TGF*β*, 20 ng/mL IL-6, 10 ng/mL IL-1*β*, 10 ng/mL IL-23, 10 μg/mL anti-IL-4, 10 μg/mL anti-IFN-*γ* for 3 days.

### 2.17 Extracellular and intracellular staining and flow cytometric analysis of murine Th17 cells

For cytokine analysis, cells were re-stimulated with PMA (1 mg/mL), Ionomycin (1 mg/mL), in the presence of GolgiStop (1:800) and GolgiPlug (1:800). 2 × 10^6^ cells were surface stained using TCR-*β* (0.2 mg/mL) and CD4 (0.2 mg/mL). Dead cells were excluded using LIVE/DEAD Fixable Aqua Dead Cell Stain Kit according to the manufacturer's instructions. For intracellular staining, the cells were fixed with Cytofix Fixation Buffer, permeabilized with Perm/Wash Buffer according to the manufacturer's instructions and stained with the following antibodies: IL-17A (0.2 mg/mL), and ROR*γ*t (0.2 mg/mL). Stained cells were measured with a FACSVerse flow cytometer (BD). Data are representative of technical duplicates of three mice that were analyzed in three independent experiments (*n* = 3) and analyzed using FlowJo 10.2 software.

### 2.18 Isolation of human peripheral blood mononuclear cells (PBMCs) and naïve CD4^+^ T cells

Whole blood from healthy, anonymous donors was purchased from the Austrian Red Cross. For PBMC isolation, whole blood was diluted 1:2 with phosphate-buffered saline (PBS; 123 mmol/L NaCl + 10 mmol/L Na_2_HPO_4_ + 3 mmol/L KH_2_PO_4_ in 1000 mL ddH2O, pH 7.4) and then carefully layered upon half of the volume of Lymphopure relative to diluted blood. Density gradient centrifugation was performed at 800 × *g* for 30 min with the break turned off. The PBMC ring was carefully isolated, collected in a tube, topped up to 50 mL with PBS and centrifuged at 800 × *g* for 5 min. After repeating the washing step once, a last washing step was done at 200 × *g* for 15 min. Afterwards, the supernatant was carefully removed, and the pellet resuspended in 15–20 mL separation buffer (0.5% bovine serum albumin (BSA) + 2 mmol/L EDTA in PBS pH 7.2). After counting the PBMCs on the Vi-CELL XR Cell Viability Analyzer, naïve CD4^+^ T cells were isolated using the Naive CD4^+^ T Cell Isolation Kit II (human) according to the manufacturer's instructions.

### 2.19 Human Th17 differentiation and flow cytometric analysis

For human Th17 differentiation, 2 × 10^5^ naïve CD4^+^ T cells were seeded per well of a 96-well plate that had been coated with *α*-CD3 antibody (5 μg/mL) the day before. Cells were polarized using 50 ng/mL IL-1*β* and 50 ng/mL IL-23 in presence of the vehicle control, the positive control or PGA and incubated for 7 days^46^. For cytokine analysis, cells were re-stimulated with the PMA/ionomycin “Cell Activation Cocktail” (PMA: 40.5 μmol/L, ionomycin: 669.3 μmol/L; 500 × ) for 2 h, and with monensin (1000 × ) for 2 additional h. Extra- and intracellular staining of Th17 cells was done using the BD Pharmingen Transcription Factor Buffer Set according to the manufacturer's instructions. Cells were stained with Fixable Viability Stain 780 (live/dead) and antibodies against CD45RO (a marker for IL-17A expressing memory T cells^47^) or CD5, and IL-17A. Flow cytometric measurements were performed on a MACSQuant Analyzer 10 and data was analyzed using FlowJo 10.8.1.

### 2.20 Sorting and flow cytometric analysis of human Th17 cells

Human CD4^+^ T cells were isolated from buffy coats purchased from the Austrian Red Cross using the EasySep™ Human CD4^+^ T Cell Isolation Kit according to manufacturer's instructions. Subsequently, cells were stained with the following antibodies: anti-human CD45 Brilliant Violet 421, anti-human CD4 FITC, anti-human CD45RO PerCP-Cy5.5, anti-human CD45RA PE, anti-human CXCR3 PE-Cy7 and anti-human CCR6 Alexa Fluor 647 and Th17 cells were isolated on a FACS Aria II Sorter (BD) according to the CD45^+^CD4^+^CD45RA^–^CD45RO^+^CXCR3^–^CCR6^+^ phenotype (purity >95% for all donors). Subsequently, 1 × 10^5^ Th17 cells were seeded per well of a 96-well plate that had been coated with *α*-CD3 antibody (5 μg/mL) the day before. Cells were treated with the vehicle control, the positive control or PGA and incubated for 3 days. Extra- and intracellular staining and flow cytometric analysis were performed as described in the previous section.

### 2.21 Mathematical and statistical analyses

Information about sample sizes, statistical analyses used, and *P* values determined for the conducted experiments are shown in the figure legends. Data are presented as mean ± standard error of the mean (SEM) or standard deviation (SD). All statistical analyses were performed using GraphPad Prism version 10 (Dotmatics, Boston, MA, USA). Significant outliers were identified and removed using Grubbs' test (*α* = 0.05). For comparing two groups, Student's two-tailed *t*-tests was performed. For comparing more than two groups, one-way ANOVA with Tukey's or Dunnett's *post hoc* test was employed. For comparing the best-fit log IC_50_ value of PGA versus another triterpenoid, an extra sum-of-squares F test was performed. Statistical significance was reached below an alpha of 0.05 in all cases. For concentration-response curve fitting and calculation of IC_50_ values, the function “log (inhibitor) *vs.* response (three parameters)” in GraphPad Prism was used. This constrained the Hill slope to a value of −1.0. For the % inhibition was calculated according to Eq. (2):

(2) $$\text{Inhibition}\;\left(\%\right)=\left(1-\frac{\text{Bottom}}{\text{Top}}\right)\times 100$$

Where “top” corresponds to the averaged baseline activity measured at the lowest tested concentration and “bottom” to the averaged residual activity measured at the highest tested concentration.

## 3 Results

### 3.1 PGA is a new oleanane-type triterpenoid RORγ inverse agonist with high potency and efficacy

**Figure 1:**
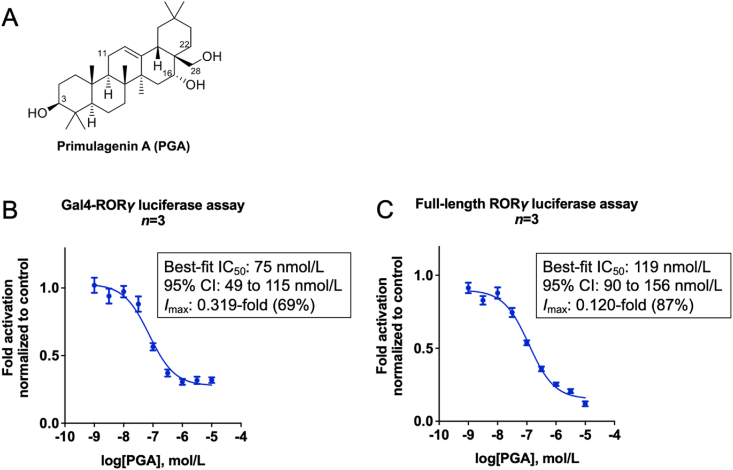
PGA acts as a highly potent and efficacious ROR*γ* inverse agonist. (A) Chemical structure of primulagenin A (PGA). (B) Concentration-response curve of PGA showing a concentration-dependent inhibition of the transactivation activity of RAR-related orphan receptor *γ* (ROR*γ*) in a Gal4-ROR*γ* luciferase assay. Human embryonic kidney (HEK) 293 cells were co-transfected with Gal4-ROR*γ*, an upstream activating sequence luciferase reporter, and eGFP. The cells were then treated with PGA at the indicated concentration. The RLU/RFU ratio was measured after an incubation time of 18 h and normalized to the vehicle control. Mean ± SEM, *n* = 3 in technical quadruplicates. (C) Concentration–response curve of PGA showing a concentration-dependent inhibition of the transactivation activity of ROR*γ* in a full-length ROR*γ* luciferase assay. HEK293 cells were co-transfected with full-length ROR*γ*, a luciferase reporter under the control of ROR response element, and eGFP, and treated with PGA at the indicated concentration. The relative luminescence unit/relative fluorescence unit ratio was measured after an incubation time of 18 h and normalized to the vehicle control. Mean ± SEM, *n* = 3 in technical quadruplicates.

Due to its structural similarity to the known inverse agonist OA, PGA (Fig. 1A and Supporting Information Fig. S1) was probed for its ability to inhibit ROR*γ* in a Gal4 luciferase assay. Fig. 1B depicts the results of the initial screening. PGA led to a concentration-dependent decrease in transactivation activity of Gal4-ROR*γ* in HEK293 cells and yielded an IC_50_ value of approx. 75 nmol/L and an *I*_max_ of 0.319-fold (69% inhibition) at 10 μmol/L. These results were confirmed using a full-length ROR*γ* luciferase assay (Fig. 1C), in which PGA showed an IC_50_ value of 119 nmol/L and an *I*_max_ value of 0.120-fold (87% inhibition) at 10 μmol/L. These results are comparable to those observed with the synthetic positive control SR2211^25^ (Supporting Information Fig. S2).

To exclude the possibility that cytotoxicity interfered with the results, resazurin conversion assays in HEK293 cells were performed, which revealed no cytotoxic effects (Supporting Information Fig. S3A). Finally, additional NR-luciferase assays were conducted to confirm the selectivity of PGA for ROR*γ* over other NRs. The following NRs were chosen for selectivity screenings as they are targeted by published triterpenoid ROR*γ* inverse agonists like UA and OA: farnesoid X receptor (FXR), liver X receptor (LXR)*α*/*β*, peroxisome proliferator–activated receptor (PPAR)*β*/*γ*, murine retinoic acid receptor (mRAR)*α*, and retinoid X receptor (RXR)*α*/*β* (reviewed in^48^^,^^49^). To assess the potential selectivity of PGA within the ROR subfamily, it was tested on ROR*α* and ROR*β* as well. Off-target effects of PGA were not detected on any of the receptors tested (Supporting Information Fig. S4). To summarize, PGA was identified as a new triterpenoid ROR*γ* inverse agonist with high potency and efficacy in cellular *in vitro* luciferase assays. Cytotoxicity was not detected, and PGA showed selectivity for ROR*γ* over other NRs usually co-modulated by triterpenoids and over ROR*α* and ROR*β*.

### 3.2 PGA is accessible in large quantities and high purity *via* isolation from Primulae radix or by organic synthesis

**Figure 2:**
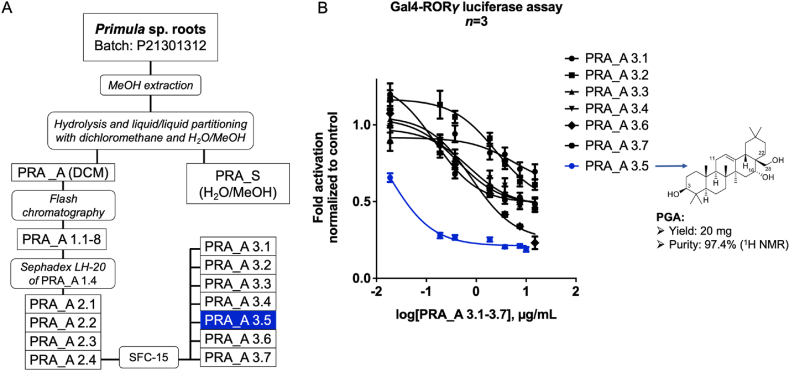
PGA was isolated from Primulae radix in high quantity and purity. (A) Simplified overview of the isolation process. (B) Concentration–response curve of the subfractions PRA_A 3.1–3.7 reveal that PRA_A 3.5 (later identified as PGA) has the highest inhibitory activity on ROR*γ* in a Gal4-ROR*γ* luciferase assay. HEK293 cells were co-transfected with Gal4-ROR*γ*, an UAS luciferase reporter, and eGFP, and treated with the extracts at the indicated concentration. The RLU/RFU ratio was measured after an incubation time of 18 h and normalized to the vehicle control. Mean ± SEM, *n* = 3 in technical quadruplicates.

For further pharmacological profiling, PGA was isolated from a suitable natural source, as it was not commercially available. Therefore, the roots of *Primula* sp. were selected for large-scale phytochemical processing and subsequent isolation of PGA. A simplified overview of this process is depicted in Fig. 2A. A hydrolyzed and sapogenin-enriched extract of Primulae radix (PRA_A) was fractionated *via* flash chromatography and size exclusion chromatography to obtain PRA_A 2.4, which was further subjected to semi-preparative supercritical fluid chromatography (SFC-15) to obtain seven subfractions (PRA_A 3.1–3.7). The bio-guided phytochemical workup pointed towards PRA_A 3.5 as the major ROR*γ* inhibiting component (Fig. 2B), which was identified as PGA with a purity of >95% (Supporting Information Table S10). Interestingly, it was found that the methanolic Primulae Radix extract, from which PGA was isolated after hydrolysis, and a commercially available cough syrup share a highly similar saponin profile (data not shown).

To attain an alternate pathway towards PGA, a synthetic route starting from commercially available echinocystic acid was established (described in “Synthesis of primulagenin A”; ^1^H and ^13^C NMR spectra are shown in Supporting Information Fig. S5). The identity and purity of both the isolated and synthesized PGA were confirmed (Table S10) and thus used for all subsequent experiments. In conclusion, two independent routes to replenish PGA for further biological studies were successfully established.

### 3.3 Structure–activity relationship of PGA and related oleanane-type triterpenoids reveals the importance of C-16 and C-28 for the activity of these compounds

**Figure 3:**
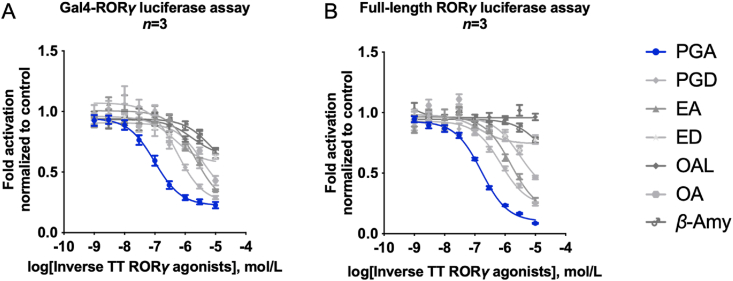
Differing activities of PGA and structurally related triterpenoids as ROR*γ* inverse agonists. (A) Concentration-response curves of PGA and structurally related triterpenoids using a Gal4-ROR*γ* luciferase assay. HEK293 cells were co-transfected with Gal4-ROR*γ*, an UAS luciferase reporter, and eGFP, and treated with the triterpenoids at the indicated concentration. The RLU/RFU ratio was measured after an incubation time of 18 h and normalized to the vehicle control. Mean ± SEM, *n* = 3 in technical quadruplicates. (B) Concentration–response curves of PGA and structurally related triterpenoids using a full-length ROR*γ* luciferase assay. HEK293 cells were co-transfected with full-length ROR*γ*, a luciferase reporter under the control of ROR response element, and eGFP, and treated with the triterpenoids at the indicated concentration. The RLU/RFU ratio was measured after an incubation time of 18 h and normalized to the vehicle control. Mean ± SEM, *n* = 3 in technical quadruplicates.

Compared to the potency and efficacy of PGA, the reported inverse agonistic activity of the triterpenoid OA on ROR*γ* (IC_50_ = 8.589 μmol/L in a luciferase assay^50^) seemed underwhelming. Hence, the luciferase assay for OA was repeated and confirmed previous findings (Table 1). Both, PGA, and OA are oleanane-type triterpenoids that share a high structural similarity. They only differ with respect to the oxidation state on C-atom 28 (–CH_2_OH (PGA) *vs*. –COOH (OA); Table 1 – “R1”) and C-atom 16 (–OH (PGA) *vs*.–H (OA); Table 1 – “R2”). A lower oxidation state of R1 and the presence of an oxygen atom on R2 appeared to be the critical factors for the superior activity of PGA over OA. To confirm this hypothesis, structurally related triterpenoids from our in-house pure compound database were tested that differed in these groups in Gal4-ROR*γ* and full-length ROR*γ* luciferase assays. Primulagenin D (PGD; R1 = –CHO, R2 = –OH), echinocystic acid (EA; R1 = –COOH, R2 = –OH), and *β*-amyrin (*β*-Amy; R1 = –CH_3_, R2 = –H) were selected to complement the data. Indeed, an increase in the oxidation state of R1 from an alcohol (PGA) to an aldehyde (PGD) to a carboxylic acid moiety (EA) led to a consistent decrease in potency and efficacy (Table 1, Fig. 3A and B).

**Table 1:** Structure–activity relationship of primulagenin A (PGA), primulagenin D (PGD), echinocystic acid (EA), erythrodiol (ED), oleanolic aldehyde (OAL), oleanolic acid (OA), and *β*-amyrin (*β*-Amy). IC_50_ and *I*_max_ values of these triterpenoids are depicted. Data were collected using Gal4-ROR*γ* and full-length ROR*γ* luciferase assays. *n* = 3 in all cases.



Tri-terpenoid	R^1^	R^2^	Best-fit IC_50_ (μmol/L)	IC_50_ 95% CI (μmol/L)	*I*_max_ at 10 μmol/L

Gal4-ROR*γ* luciferase assay					
PGA	–CH_2_OH	–OH	0.099	0.068–0.143	0.228-fold (75% inh.)
PGD	–CHO	–OH	0.542	0.335 – 0.889	0.290-fold (71% inh.)
EA	–COOH	–OH	2.127	1.082 – 4.474	0.349-fold (62% inh.)
ED	–CH_2_OH	–H	0.456	0.214 – 0.968	0.627-fold (33% inh.)
OAL	–CHO	–H	1.739	0.381 – 21.300	0.653-fold (29% inh.)
OA	–COOH	–H	3.625	0.922 – 27.260	0.429-fold (54% inh.)
*β*-Amy	–CH_3_	–H	6.666	1.105 – n.d.	0.674-fold (29% inh.)
Full-length ROR*γ* luciferase assay					
PGA	–CH_2_OH	–OH	0.165	0.139 – 0.197	0.086-fold (91% inh.)
PGD	–CHO	–OH	0.717	0.463 – 1.113	0.246-fold (75% inh.)
EA	–COOH	–OH	1.556	1.010 – 2.436	0.275-fold (68% inh.)
ED	–CH_2_OH	–H	0.143	0.037 – 0.622	0.778-fold (21% inh.)
OAL	–CHO	–H	Ambiguous	Ambiguous	0.964-fold (6% inh.)
OA	–COOH	–H	2.988	1.033 – 10.230	0.464-fold (47% inh.)
*β*-Amy	–CH_3_	–H	14.770	1.128 – n.d.	0.775-fold (18% inh.)

The lack of an –OH group on R2 further decreased both potency and efficacy (*e.g*., EA *vs.* OA, Table 1, Fig. 3A and B). Lastly, a CH_3_-group on R1 and an H-atom on R2 (*β*-Amy) abolished ROR*γ* activity (Table 1, Fig. 3A and B). Interestingly, erythrodiol (ED; R1=CH_2_OH, R2 = –H; synthesis described in “Synthesis of erythrodiol” and ^1^H- and ^13^C-NMR spectra shown in Supporting Information Fig. S6), showed a favorable potency but a loss of efficacy (Table 1, Fig. 3A and B), underpinning the importance of an –OH group on R2. This was even more pronounced in case of the newly synthesized compound oleanolic aldehyde (OAL; R1 = –CHO, R2 = –H; synthesis described in “Synthesis of oleanolic aldehyde” and ^1^H- and ^13^C-NMR spectra shown in Supporting Information Fig. S7), which was completely inactive on full-length ROR*γ* (Table 1, Fig. 3A and B). To test whether the superiority of PGA was statistically significant, we compared the best-fit log IC_50_ values of PGA with all other structurally related triterpenoids listed in Table 1 using an extra sum-of-squares F test. Indeed, PGA was statistically significantly different from the other triterpenoids in terms of its log IC_50_ value, except for ED in the full-length luciferase assay (Supporting Information Table S11). However, a comparison of the best-fit bottom values of the concentration-response curves revealed a significant difference between PGA and ED (data not shown), corroborating that while their potencies are similar, the efficacy of PGA is superior. Importantly, the difference in the log IC_50_ value of PGA and the highly active ursane-type triterpenoid ROR*γ* inverse agonist UA was confirmed to be statistically significant as well^51^ (Supporting Information Fig. S8). No cytotoxic effects of any of the compounds at 10 μmol/L (Fig. S3B) were observed, and we confirmed a high purity for all of them (see Table S10 for isolated or synthesized triterpenoids and Table S1 for commercially obtained ones). All chemical structures discussed in this work are summarized in Fig. S1. Taken together, a low oxidation state on R1 combined with the presence of an –OH group on R2 led to the highest inverse agonistic activity of oleanane-type triterpenoids on ROR*γ*. Moreover, it was shown for the first time that the oleanane-type triterpenoids PGD, EA, and ED are also inverse agonists of ROR*γ*.

### 3.4 Molecular docking and site-directed mutagenesis suggest unique and distinct protein–ligand interaction patterns for PGA and OA

**Figure 4:**
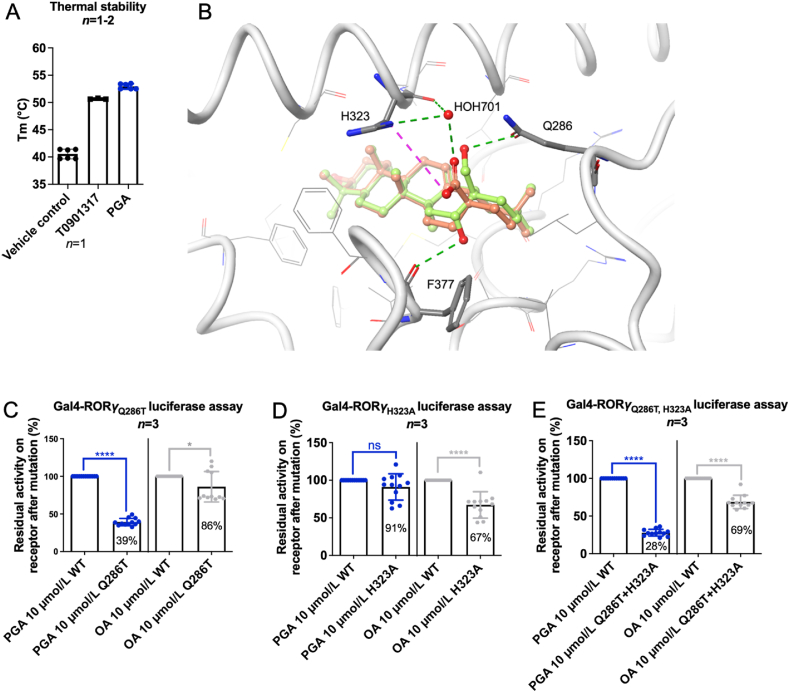
PGA directly binds to the hROR*γ* LBD, and both PGA and OA are predicted to form partially distinct protein–ligand interactions with the ROR*γ* LBD. (A) Thermal stabilization upon binding of T0901317 and PGA to the hROR*γ* LBD. Nano differential scanning fluorimetry was used to compare the thermal stability of the purified apo hROR*γ* LBD and the ligand-bound protein. Mean ± SD, *n* = 1–2 in technical triplicates. (B) Docking pose illustrating how PGA (green carbon atoms) and OA (orange carbon atoms) are expected to bind to the human ROR*γ* LBD (PDB structure: 6J3N, in gray carbon atoms and tube representation). Residues predicted to engage in important interactions with the ligands are marked and highlighted by thick tube representations. Predicted hydrogen bonds are indicated by green dashed lines, and the salt bridge by magenta dashed lines. (C–E) ROR*γ*_mutant_-Gal4 luciferase assays showing the differences in activity between PGA or OA on the WT NR (100% activity) *vs.* the respective mutant NR (% residual activity). HEK293 cells were co-transfected with the Gal4-ROR*γ* WT or a mutant, an UAS luciferase reporter, and eGFP, and treated with PGA or OA at 10 μmol/L. The RLU/RFU ratio was measured after an incubation time of 18 h and normalized to the vehicle control. Mean ± SD, *n* = 3 in technical quadruplicates. Student's two-tailed *t*-test was performed for statistical analysis. ∗∗∗∗*P* ≤ 0.0001, ∗*P* ≤ 0.05, ns *P* > 0.05.

First, to verify direct binding of PGA to the human ROR*γ* (hROR*γ*) LBD, nano differential scanning fluorimetry (nanoDSF) was employed^52^. As shown in Fig. 4A, PGA stabilized the hROR*γ* LBD to a greater extent (melting temperature of approx. 53.0 °C) compared to the ROR*γ* inverse agonist T0901317 (approx. 50.7 °C)^53^. Next, to understand the structural basis for the differing levels of potency and efficacy between PGA and the published oleanane-type triterpenoid OA on ROR*γ*, docking and site-directed mutagenesis experiments were performed. Fig. 4B shows the docking poses derived for PGA and OA bound to the ROR*γ* LBD. The 28-OH group of PGA was predicted to form a direct hydrogen bond to glutamine 286 (Q286) of the ROR*γ* LBD, while OA was unlikely to form such an interaction. Consistent with this prediction, the Q286T mutation led to a significant reduction of the activity of PGA compared to its activity on the wild-type (WT) NR, while the activity of OA was only slightly (but still significantly) reduced (Fig. 4C). Notably, PGA was predicted to form an additional hydrogen bond with the backbone of F377 using its 16-OH group, a group that OA lacks.

Furthermore, OA was predicted to form a direct and indirect hydrogen bond (mediated by water molecule 701) with H323 of the ROR*γ* LBD. In contrast, PGA was predicted not to interact with this amino acid. Consistent with these predictions, the H323A mutation left the activity of PGA unaltered. However, the mutation led to a significant decrease in OA's activity on the NR compared to WT (Fig. 4D). Of note, the H323A mutation did not affect the activity of OA as much as the Q286T mutation did in the case of PGA. This suggests that the hydrogen bond formed between OA and H323 might indeed be mediated *via* water molecule 701, as predicted. The Q286T + H323A double mutant resulted in similar reductions in activity (Fig. 4E), supporting the notion that both mutations independently reduce the activity of PGA and OA, respectively, but not synergistically. The “raw” changes in fold activation after PGA or OA treatment of WT *vs.* ROR*γ* mutants are depicted in Supporting Information Fig. S9. From a molecular point of view, the higher activity of PGA compared to the published ROR*γ* inverse agonist OA is predicted to be due to hydrogen bond “anchoring” (with the backbone of F377) within the ROR*γ* LBD, made possible by the additional 16-OH group. Moreover, the energy penalty related to the desolvation of the carboxylic acid moiety in position C-28 could also be a decisive factor for the lower activity of OA compared to PGA. Both predictions align well with our observations regarding the structure–activity relationship (see previous section). Overall, direct binding of PGA to the hROR*γ* LBD was confirmed, and the likely differences in protein–ligand interaction patterns for PGA and OA within the ROR*γ* LBD were elucidated.

### 3.5 PGA downregulates the expression of RORγ target genes in different cell lines

**Figure 5:**
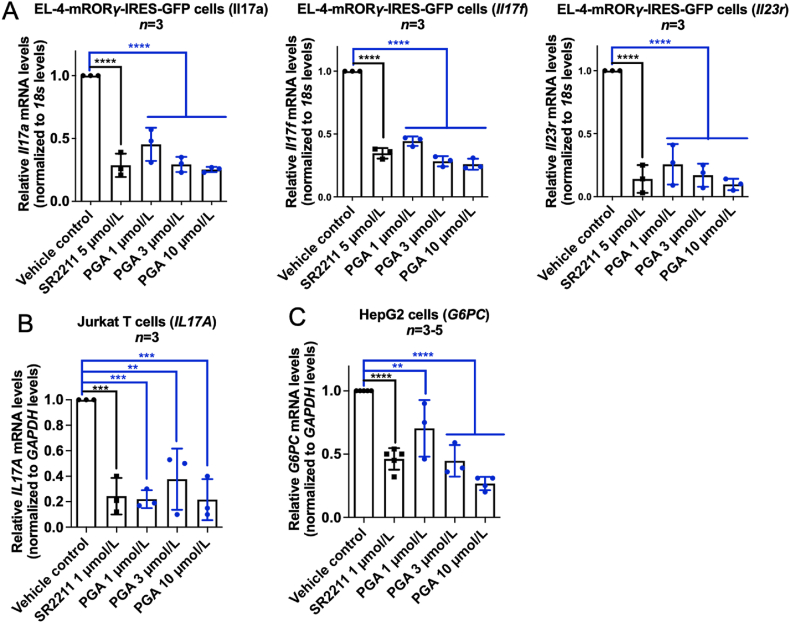
PGA decreases the expression of ROR*γ* target genes in three different cell lines. (A) Decrease of Il17a, Il17f, and Il23r expression in the EL-4-mROR*γ*t cell line upon PGA treatment. 18S was used as a control gene. Mean ± SD, *n* = 3 in technical triplicates. (B) Decrease of IL17A expression in Jurkat T cells transiently transfected with hROR*γ* upon PGA treatment. GAPDH was used as a control gene. *n* = 3 in technical triplicates. (C) Decrease of G6PC expression in HepG2 cells transiently transfected with hROR*γ* upon PGA treatment. GAPDH was used as a control gene. Mean ± SD, *n* = 5 (vehicle control, SR2211), *n* = 4 (PGA 10 μmol/L), *n* = 3 (rest) in technical triplicates. One-way ANOVA with Dunnett's test was performed for statistical analysis. ∗∗∗∗*P* ≤ 0.0001, ∗∗∗*P* ≤ 0.001, ∗∗*P* ≤ 0.01.

To examine whether PGA affects ROR*γ* target gene expression, qPCR experiments were performed using three different cell lines, HepG2, Jurkat T, and EL-4 cells. HepG2 and Jurkat T cells were transiently transfected with human ROR*γ* prior to experiments. EL-4 cells were stably transfected with murine ROR*γ* (see “Generation of the stable EL-4-mROR*γ*t cell line” for details and Supporting Information Fig. S10 for the flow cytometric gating strategy). As expected, PGA led to a significant down-regulation of the ROR*γ* target genes *Il17a/f*, *Il23r* (all of them in EL-4-mROR*γ*t cells), *IL-17A* (Jurkat T cells), and glucose-6-phosphatase (*G6PC*; HepG2 cells*)* (Fig. 5A–C) while showing no cytotoxic effect in any of the cell lines used (Fig. S3C–S3E). In brief, PGA led to a decreased expression of ROR*γ* target genes in three different cell lines overexpressing this NR.

### 3.6 PGA inhibits the differentiation of murine and human CD4^+^ T cells into Th17 cells and inhibits the release of IL-17A from pre-differentiated human Th17 cells

**Figure 6:**
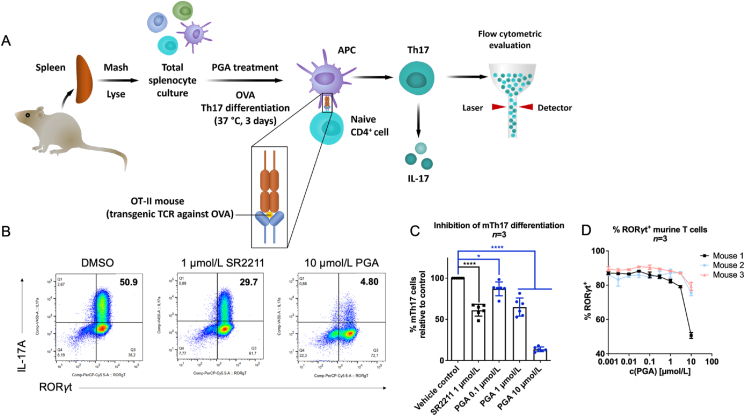
PGA concentration-dependently inhibits murine T helper (Th)17 differentiation. (A) Schematic overview of the experimental setup measuring murine Th17 differentiation. (B) Flow cytometric analysis of ovalbumin-specific CD4^+^ T-cells activated with ovalbumin peptide and cultured under Th17-polarizing conditions in the presence or absence of PGA for three days. Numbers indicate the percentage of interleukin (IL)-17A^+^/ROR*γ*t ^+^ cells within viable CD4^+^ T cells. (C) Bar graph showing the percentages of ROR*γ*t^+^/IL-17A^+^ CD4^+^ T cells after treatment with SR2211 or PGA relative to vehicle control treatment. Mean ± SD, *n* = 3 in technical duplicates. One-way ANOVA with Dunnett's test was performed for statistical analysis. ∗∗∗∗*P* ≤ 0.0001, ∗*P* ≤ 0.05. (D) The percentage of viable ROR*γ*t ^+^ cells upon PGA treatment.

As a next step, it was assessed whether PGA affects murine and human Th17 differentiation**.** For murine Th17 cells, total splenocytes of OT-II transgenic mice were cultured under Th17 polarizing conditions for three days and simultaneously treated with the vehicle control, SR2211 (1 μmol/L) or PGA at different concentrations. Fig. 6A shows the workflow while Fig. 6B depicts the flow cytometric analysis with the percentage of the IL-17A/ROR*γ*t double-positive population (IL-17^+^/ROR*γ*t^+^) after treatment (the complete gating strategy is depicted in Supporting Information Fig. S11). PGA treatment led to a concentration-dependent decrease of IL-17^+^/ROR*γ*t ^+^ cells (Fig. 6C). While PGA significantly reduced IL-17A expression, the overall percentage of ROR*γ*t ^+^ cells remained over 75% at all PGA concentrations tested and above 85% at 3 μmol/L and at lower concentrations (Fig. 6D) except for mouse 1 (Fig. 6D, black curve) where a decrease in the percentage of ROR*γ*^+^ cells to approximately 50% was observed at 10 μmol/L. This drop coincides with a decrease in cell viability at 10 μmol/L (Supporting Information Fig. S12) and could therefore be non-specific, although the percentage of ROR*γ*t^+^ (and IL-17A^+^) cells reflect only the viable population (see gating strategy in Fig. S11). Thus, this phenomenon could be an experimental artifact.

**Figure 7:**
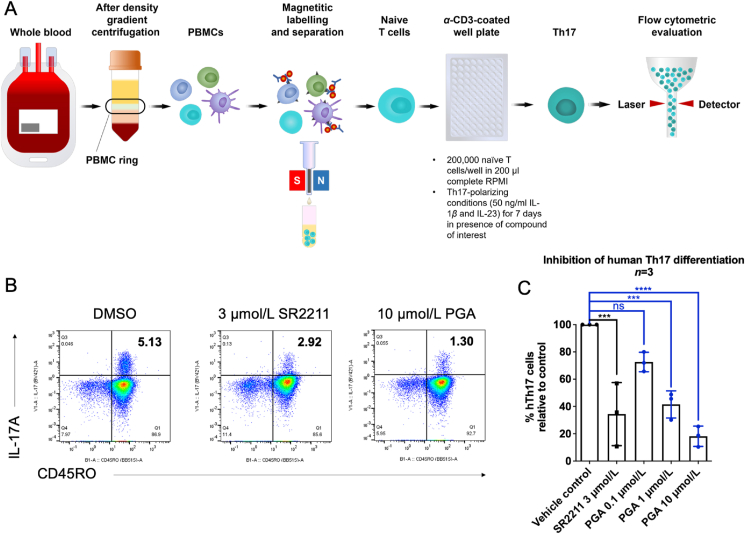
PGA concentration-dependently inhibits human Th17 differentiation. (A) Schematic overview of the experimental setup involving human Th17 differentiation. (B) Flow cytometric analysis of naïve CD4^+^ T-cells activated with plate-bound *α*-CD3 antibody and cultured under Th17-polarizing conditions in the presence or absence of PGA for seven days. Numbers indicate the percentage of CD45RO^+^ (a marker for IL-17A expressing memory T cells^47^) or CD5^+^/IL-17A^+^ cells. (C) Bar graph showing the percentages of CD45RO^+^ or CD5^+^/IL-17A^+^ helper T cells after treatment with SR2211 or PGA relative to vehicle control treatment. Mean ± SD, *n* = 3 (three healthy, anonymous donors). One-way ANOVA with Dunnett's test was performed for statistical analysis. ∗∗∗∗*P* ≤ 0.0001, ∗∗∗*P* ≤ 0.001.

Next, naïve human CD4^+^ T cells isolated PBMCs of healthy anonymous donors were differentiated toward Th17 cells for seven days^46^ and simultaneously treated with compounds of interest. Fig. 7A represents the experimental workflow, while Fig. 7B depicts dot plots of the flow cytometric analysis, which shows that SR2211 (3 μmol/L) and PGA (10 μmol/L) inhibit the differentiation of human Th17 cells compared to vehicle control (the complete gating strategy is shown in Supporting Information Fig. S13). A concentration-dependent decrease in the percentage of human Th17 cells upon PGA treatment (Fig. 7C) was observed, confirming the findings obtained from murine cells.

**Figure 8:**
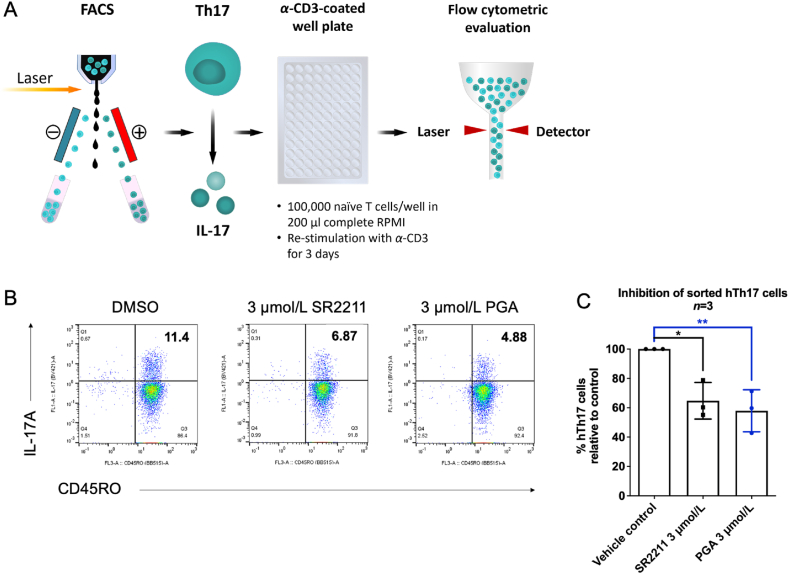
(A) Schematic overview of the experimental setup using isolated human Th17 cells. (B) Flow cytometric analysis (showing IL-17A and CD45RO) of sorted Th17 cells re-stimulated with plate-bound *α*-CD3 antibody and cultured in the presence or absence of PGA for three days. Numbers indicate the percentage of cells in the upper right quadrant. (C) Bar graph showing the percentages of CD45RO/IL-17A^+^ CD4^+^ T cells after treatment with SR2211 or PGA relative to vehicle control treatment. Mean ± SD, *n* = 3 (three healthy, anonymous donors). One-way ANOVA with Dunnett's test was performed for statistical analysis. ∗∗*P* ≤ 0.01, ∗*P* ≤ 0.05.

Finally, the influence of PGA on already differentiated human Th17 cells was explored. To this end, human Th17 cells (CD4^+^CD45RO^+^CXCR3^–^CCR6^+^; Supporting Information Fig. S14) were isolated by fluorescence-activated cell sorting (FACS) from enriched CD4^+^ T cells isolated from buffy coats of healthy anonymous donors. Isolated cells were re-stimulated with plate-bound *α*-CD3 and simultaneously treated with the respective compounds for three days. The workflow is depicted in Fig. 8A. Dot plots in Fig. 8B show that SR2211 (3 μmol/L) and PGA (3 μmol/L) reduced the number of IL-17A-producing cells compared to the vehicle control. PGA led to a similar reduction in IL-17A^+^ cells compared to the positive control SR2211 (Fig. 8C).

Collectively, PGA inhibited both murine and human Th17 differentiation in a concentration-dependent manner. Moreover, it was able to decrease the percentage of IL-17A producing cells from already differentiated Th17 cells at 3 μmol/L.

## 4 Discussion

By using a combination of *in vitro*, *in silico*, and *ex vivo* approaches, the oleanane-type triterpenoid PGA, derived from the traditionally used herbal remedy Primulae radix was unveiled as a potent and efficacious inverse agonist of the NR ROR*γ*, the main transcription factor for the differentiation of pro-inflammatory Th17 cells^18^. PGA showed a high potency and efficacy in luciferase assays (IC_50_ approx. 100 nmol/L, *I*_max_ up to 91%), concentration-dependently reduced ROR*γ* target gene expression in different cell lines and inhibited murine and human Th17 cell differentiation. Additionally, it was able to decrease the percentage of IL-17-producing cells in sorted human Th17 cells, which is consistent with the role of ROR*γ*t as a “safeguard” of the Th17 lineage^54^.

PGA is a sapogenin obtained by hydrolysis and phytochemical processing of native Primula saponins containing the aglycone protoprimulagenin A.^28^ The traditional and, to some extent, scientifically validated^10^, ^11^, ^12^, ^13^ respiratory effects of Primulae radix may at least partly be explained by inhibition of ROR*γ*, a target that has been linked to respiratory disorder treatment as well^55^. This hypothesis is further supported by the high similarity between the saponin profiles of the plant extract and a commercially available cough syrup. However, it remains unclear whether the acidic environment in the stomach is sufficient to facilitate the hydrolysis of these saponins and the subsequent formation of PGA^56^. This aspect warrants further investigation.

The identified natural product PGA stands out among other known small-molecule inverse agonists of ROR*γ* by its promising potency, suggesting potential therapeutic relevance in the context of Th17-mediated diseases, including psoriasis, psoriatic arthritis, RA, and IBD. Although antibodies against IL-17, like secukinumab, are well-established therapeutics for psoriasis and psoriatic arthritis, they surprisingly either show low efficacy or even increase disease severity in RA and IBD, respectively^19^. One explanation could be that Th17 cells change their phenotype from predominantly producing IL-17 to predominantly producing interferon (IFN)-*γ* in the course of these diseases. Due to a lack of IL-17 production from such ex-Th17 (or nonclassical Th1) cells, anti-IL-17 antibodies are ineffective^57^. Th17 cells have been referred to as “moving targets” due to this plasticity and their ability to alter the cytokines they release^58^. The ensuing therapeutic “loophole” might be effectively closed by employing inhibitors that target ROR*γ*t and thus their differentiation rather than the cytokines they secrete. Furthermore, small molecules such as PGA offer advantages such as lower costs and the possibility of oral administration compared to antibodies. The latter point is highly dependent on the pharmacokinetic profile, which is currently unknown for PGA. Given the structural similarity between PGA and OA, it is plausible that PGA (like OA) has low bioavailability^59^. However, semisynthetic derivatives of OA with improved bioavailability have been developed^60^, a strategy that can also be applied to PGA. To generate such derivatives, *in silico*-guided semisynthetic modifications of PGA will be utilized. A first step towards this has already been taken in this study, i) by confirming direct binding of PGA to the hROR*γ* LBD and ii) by unveiling the likely binding mode of PGA within the ROR*γ* LBD by molecular docking. However, predictions made by docking still need additional experimental verification, especially since the backbone interaction with F377 could not be verified using site-directed mutagenesis. To further address this, crystallographic attempts were made. While these yielded crystals, the low electron density in the ligand-binding pocket prevented precise ligand placement (data not shown), underscoring the need for future high-resolution structural studies to confirm the binding mode.

The most pressing issue, which potentially limits the therapeutic use of PGA, originates from the uncertain safety profile of ROR*γ* inverse agonists. While the findings collected in this study demonstrate the non-cytotoxicity of PGA at the used concentrations, the general applicability of ROR*γ* inverse agonists as therapeutics has been questioned. It was shown that *Rorc*^*−/−*^ mice have a high incidence of metastatic thymic lymphomas that tend to metastasize into the spleen and liver^61^. The development of lymphoblastic lymphomas was also observed in ROR*γ* adult induced knockout mice^62^. Importantly, treatment of rasH2-Tg hemizygous mice with the ROR*γ* inverse agonist BMS-986251 effectively phenocopied *Rorc*^−/−^ mice by leading to a high incidence of thymic lymphomas and the discontinuation of this inhibitor^63^. All these findings raise substantial safety concerns when it comes to the therapeutic use of ROR*γ* inverse agonists. In this regard, ROR*α* was suggested as a “safer” therapeutic target compared to ROR*γ* since its inhibition did not cause thymic apoptosis (which was linked to the development of lymphomas^61^) but still suppressed Th17 differentiation and the development and severity of experimental autoimmune encephalomyelitis, a disease model for MS^47^. On the other hand, the ROR*γ* inverse agonist IMU-935 was deemed safe with no dose-limiting toxicities in a double-blind, placebo-controlled, first-in-human phase 1 study^64^. Prior to that study, authors demonstrated that IMU-935 had no effect on thymocyte maturation *in vitro* and did not completely inhibit ROR*γ*. Instead, it maintained the receptor at approximately 20% of its basal activity in a Gal4-based luciferase assay at the highest concentration used^65^. This suggests that the remaining activity of ROR*γ* is sufficient to support proper thymocyte maturation. Thus, by employing “partial” inverse agonists like IMU-935, detrimental side effects of ROR*γ* inhibition could be avoided^66^. In comparison, 10 μmol/L PGA leaves approximately 30% and 10% of the basal ROR*γ* activity in the Gal4 and full-length luciferase assay, respectively. While this corresponds well to the luciferase data collected for IMU-935, it is ultimately no proof for the safety of PGA.

Despite the significant findings and promising profile of PGA, the current study has limitations that offer directions for further research. One limitation is the absence of *in vivo* validation. While the presented *in vitro* and *ex vivo* data provide a robust mechanistic foundation, the therapeutic efficacy or safety of PGA in complex autoimmune models such as EAE remains to be demonstrated. Furthermore, although extensive biophysical, computational, and molecular biology data support the proposed binding mechanism, obtaining direct high-resolution structural evidence remains an important future goal to confirm the precise interaction patterns. Lastly, this investigation was primarily focused on Th17 cells, and a broader immunological profiling of PGA's effects on other T cell subsets is needed for a more complete understanding of its immunomodulatory potential. Ultimately, this study identifies PGA as a key hit scaffold, and addressing these points represents a critical next step, ideally pursued using optimized PGA analogues instead of PGA itself.

In summary, PGA, an oleanane-type triterpenoid from the traditionally used herbal medicine Primulae Radix was discovered as a new ROR*γ* inverse agonist with high potency and efficacy.

## Author contributions

This study was conceptualized by Patrik F. Schwarz, Alexander F. Perhal, and Verena M. Dirsch. Verena M. Dirsch provided overall supervision and resources for the study. Alexander F. Perhal offered mentorship and ongoing guidance. Compounds were synthesized by Jasmin Janneschütz under the supervision of Nina Schützenmeister, who also provided the resources for the syntheses. PGA was isolated under the supervision of Ulrike Grienke and Judith M. Rollinger. Compounds were analyzed by Jasmin Janneschütz, Nina Schützenmeister, and Ulrike Grienke. Biological experiments were performed and analyzed by Patrik F. Schwarz, Alexander F. Perhal, Teresa Preglej, and Lina Breit. Experiments regarding nanoDSF were conducted under the supervision of Natacha Rochel. Computational methods were executed by Ya Chen and supervised by Johannes Kirchmair. Experiments regarding mouse Th17 cells were conducted by Lina Breit under the supervision of Teresa Preglej and Michael Bonelli, the latter also provided the resources for these experiments. Experiments regarding sorting of human Th17 cells were conducted by Klaus G. Schmetterer, who also provided the resources for these experiments. Visualization was performed by Patrik F. Schwarz, Teresa Preglej, Lina Breit, Klaus G. Schmetterer, and Ya Chen. Patrik F. Schwarz wrote the original draft of the manuscript; Alexander F. Perhal and Verena M. Dirsch revised it. Contributions to the original draft were made by Teresa Preglej, Klaus G. Schmetterer, Ulrike Grienke, Judith M. Rollinger, Jasmin Janneschütz, Nina Schützenmeister, Ya Chen, and Johannes Kirchmair. Thereafter, all authors reviewed and edited the manuscript. All authors have read and agreed to the published version of the manuscript.

## Declaration of generative AI and AI-assisted technologies in the writing process

During the preparation of this work, authors used AI to improve the readability and language of the manuscript. After using these tools, authors reviewed and edited the content as needed and therefore take full responsibility for the content of the published article.

## Conflicts of interest

The authors declare no conflicts of interest.

## Appendix A Supporting information

The following is the Supporting information to this article:
